# Evaluation of agonist and antagonist radioligands for somatostatin receptor imaging of breast cancer using positron emission tomography

**DOI:** 10.1186/s41181-017-0023-y

**Published:** 2017-04-17

**Authors:** Iulia Dude, Zhengxing Zhang, Julie Rousseau, Navjit Hundal-Jabal, Nadine Colpo, Helen Merkens, Kuo-Shyan Lin, François Bénard

**Affiliations:** 1 Department of Molecular Oncology, BC Cancer Agency Research Centre, 675 West 10th Ave, Vancouver, V5Z 1 L3 BC Canada; 2 Department of Radiology, University of British Columbia, Vancouver, BC Canada

**Keywords:** Somatostatin receptor, Breast cancer, Antagonists, Positron emission tomography, Peptides, JR11, ZR-75-1

## Abstract

**Background:**

The somatostatin receptor subtype 2 (sstr2) is expressed on a majority of luminal breast cancers, however SPECT and scintigraphy imaging with agonistic sstr2 probes has been sub-optimal. High affinity antagonists can access more binding sites on the cell surface, resulting in higher tumor uptake and improved sensitivity. We compared the tumor uptake and biodistribution of the antagonist ^68^Ga-NODAGA-JR11 with two agonists ^68^Ga-DOTA-Tyr^3^-octreotide (^68^Ga-DOTATOC) and ^68^Ga-DOTA-Tyr^3^-octreotate (^68^Ga-DOTATATE), in the human, sstr2-positive, luminal breast cancer model: ZR-75-1.

**Results:**

Peptides were assayed for binding affinity using a filtration-based competitive assay to sstr2. ^nat^Ga-DOTATOC and ^nat^Ga-DOTATATE had excellent affinity (inhibition constant K_i_: 0.9 ± 0.1 nM and 1.4 ± 0.3 nM respectively) compared to ^nat^Ga-NODAGA-JR11 (25.9 ± 0.2 nM). The number of binding sites on ZR-75-1 cells was determined in vitro by saturation assays. Agonist ^67/nat^Ga-DOTATOC bound to 6.64 ± 0.39 × 10^4^ sites/cells, which was 1.5-fold higher than ^67/nat^Ga-NODAGA-JR11 and 2.3-fold higher than ^67/nat^Ga-DOTATATE. All three ^68^Ga-labeled peptides were obtained in good decay-corrected radiochemical yield (61-68%) and were purified by high performance liquid chromatography to ensure high specific activity (137 – 281 MBq/nmol at the end of synthesis). NOD *scid* gamma mice bearing ZR-75-1 tumors were injected intravenously with the labeled peptides and used for PET/CT imaging and biodistribution at 1 h post-injection. We found that ^68^Ga-DOTATOC had the highest tumor uptake (18.4 ± 2.9%ID/g), followed by ^68^Ga-DOTATATE (15.2 ± 2.2%ID/g) and ^68^Ga-NODAGA-JR11 (12.2 ± 0.8%ID/g). Tumor-to-blood and tumor-to-muscle ratios were also higher for the agonists (>40 and >150 respectively), compared to the antagonist (15.6 ± 2.2 and 45.2 ± 11.6 respectively).

**Conclusions:**

The antagonist ^68^Ga-NODAGA-JR11 had the lowest tumor uptake and contrast compared to agonists ^68^Ga-DOTATOC and ^68^Ga-DOTATATE in ZR-75-1 xenografts.

The main contributing factor to this result could be the use of an endogenously expressing cell line, which may differ from previously published transfected models in the number of low-affinity, antagonist-specific binding sites. The relative merit of agonists versus antagonists for sstr2 breast cancer imaging warrants further investigation, first in preclinical models with other sstr2-positive breast cancer xenografts, and ultimately in luminal breast cancer patients.

**Electronic supplementary material:**

The online version of this article (doi:10.1186/s41181-017-0023-y) contains supplementary material, which is available to authorized users.

## Background

The somatostatin family of G-protein coupled receptors is comprised of five different subtypes which are variably expressed on many cancer types, most notably neuroendocrine tumors (NETs). Somatostatin receptor subtype 2 (sstr2) is the most commonly overexpressed subtype, and hence several high-affinity radiolabeled peptides (mostly agonists) have been developed for this target (Fani et al. 2012a; Krenning et al. 1993; Kwekkeboom et al. 2010; Maecke & Reubi 2011; Reubi et al. 2001; Reubi 2003). Such tracers have been used for diagnosis, as is the case with ^111^In-DTPA-D-Phe-octreotide (^111^In-pentatreotide), ^68^Ga-DOTA-Tyr^3^-octreotate (^68^Ga-DOTATATE) and ^68^Ga-DOTA-Tyr^3^-octreotide (^68^Ga-DOTATOC), or therapy with ^90^Y-DOTATOC and ^177^Lu-DOTATATE (Fani et al. 2012a; Krenning et al. 1993; Kwekkeboom et al. 2010; Maecke & Reubi 2011; Reubi 2003).

Similar to NETs, breast tumors differentially express somatostatin receptors compared to non-malignant tissue (Fani et al. 2012b). Several studies have evaluated the expression of sstr2 in different patient cohorts, showing that 15-66% of breast tumors were sstr-positive by autoradiography (Dalm et al. 2015; Foekens et al. 1989; Bootsma et al. 1993), 30-85% by immunohistochemistry (Ciocca et al. 1990; Schulz et al. 1998), and 97-100% by mRNA analysis (Kumar et al. 2005; Vikic-Topic et al. 1995; Evans et al. 1997). The high variability observed between reports may be due to heterogeneous intra-tumor receptor density (Reubi et al. 1990) and lack of patient stratification. Sstr2 expression strongly correlates with luminal A markers (estrogen and progesterone receptor), and is typically not found in the other breast cancer subtypes (Dalm et al. 2015; Frati et al. 2014; Reubi & Torhorst 1989). Imaging with sstr agents can therefore be applicable to a large patient population, as luminal A cancers comprise 75% of all breast cancer cases (Kwan et al. 2009).

Compared to NETs, breast cancer sstr2 density is lower and more heterogeneous as determined using autoradiography (Reubi et al. 2002; Cescato et al. 2011) and PET/CT imaging (Elgeti et al. 2008). Diagnostic SPECT and scintigraphy with ^111^In-pentatreotide and ^99m^Tc-depreotide, have been explored clinically in patients with sstr2-positive breast cancer (Alberini et al. 2000; van Eijck et al. 1994; Wang et al. 2008). Sensitivity suffered in these initial studies, partly due to the low resolution of conventional scintigraphy, as well as the lower and more heterogeneous expression of sstr2 on breast carcinoma compared to NETs (Alberini et al. 2000; van Eijck et al. 1994; Wang et al. 2008).

More recently, a number of reports have identified several antagonist somatostatin analogs that visualized sstr2-positive tumors better than conventional agonists in both preclinical and clinical cases. Ginj et al. first demonstrated this finding by comparing an sstr3 agonist and sstr3 antagonist of similar binding affinities in a tumor of human embryotic kidney (HEK) cells transfected with sstr3. Scatchard analysis of these compounds revealed that the antagonist bound to more sites on the tumor cells, resulting in an overall higher tumor uptake, despite marginally lower binding affinity and no internalization capacity (Ginj et al. 2006). Since then, several sstr2 antagonists, labeled with either diagnostic or therapeutic isotopes, have been explored (Fani et al. 2012b; Cescato et al. 2011; Fani et al. 2011; Wild et al. 2011; Wild et al. 2014). Cescato et al. performed in vitro autoradiography on several sstr2-positive primary tumor samples, including breast carcinomas, and suggested that the antagonist ^177^Lu-DOTA-BASS bound to more sites on the tumor samples than the agonist ^177^Lu-DOTATATE (Cescato et al. 2011). In clinical studies involving NET patients, the diagnostic antagonist ^111^In-DOTA-BASS showed improved contrast compared the conventional agonist ^111^In-pentatreotide (Wild et al. 2011). Furthermore, the therapeutic antagonist ^177^Lu-DOTA-JR11 delivered a 1.7 - 10.6 fold higher tumor dose than the agonist ^177^Lu-DOTATATE in a small pilot study comprised of 4 patients with advanced NETs (Wild et al. 2014).

Several breast cancer patients with recurrent or metastatic luminal breast cancer eventually develop resistance to endocrine therapies (Milani et al. 2014). The subset of patients that express high levels of sstr2 might benefit from treatment by peptide receptor radionuclide therapy (PRRT) with somatostatin analogs if sufficient radiotracer accumulation is achieved (Dalm et al. 2016). Because antagonists can bind to more sites, it is possible that tumors with lower sstr2 density, such as breast cancers, might still be visualized and treated with radiolabeled peptides.

^68^Ga-NODAGA-JR11 showed excellent tumor uptake and biodistribution compared to ^68^Ga-DOTATATE in a preclinical setting (Fani et al. 2012b), and ~15% higher SUVmax uptake in NET patients compared to ^68^Ga-DOTATOC (Nicolas et al. 2015). The aim of this study was to compare a potent antagonist, ^68^Ga-NODAGA-JR11, and two commonly used agonists, ^68^Ga-DOTATATE and ^68^Ga-DOTATOC, for in vivo breast cancer imaging using a human xenograft model with endogenous sstr2 expression. We used the sstr2-positive luminal A breast cancer cell line ZR-75-1 (Subik et al. 2010) and determined the transcriptional expression of the five sstr subtypes in those cells. The binding affinity of the peptides to human sstr2 was measured using identical assay conditions, and biodistribution of the radiolabeled peptides was compared.

## Methods

### General methods

All reagents and solvents were purchased from commercial sources and used without further purification except otherwise specified. Peptides DOTATATE, DOTATOC, and NODAGA-JR11 and the cold standards of their natural gallium (^nat^Ga) complexes were prepared by standard fmoc solid-phase peptide synthesis according to literature procedures (Fani et al. 2012b; Heppeler et al. 1999). C18 Sep-Pak cartridges (1 cm^3^, 50 mg) were obtained from Waters Corporation (Milford, MA) and pre-washed with ethanol followed by deionized (DI) water prior to use. High performance liquid chromatography (HPLC) purification and quality control were performed on a semi-preparative column (C18, 5 μm, 250 × 10 mm), or an analytical column (C18, 5 μm, 250 × 4.6 mm) respectively, both purchased from Phenomenex (Torrance, CA) and used on an Agilent 1260 infinity platform (Santa Clara, CA). Triethylammonium phosphate buffer (TEAP), phosphate buffered saline (PBS) and acetonitile (MeCN) were used for HPLC elution. TEAP buffer (pH 7.3) was prepared by titrating triethylamine (8 mL) with o-phosphoric acid in DI water (1 L), and PBS (pH 7.4) was prepared by dissolving PBS powder or tablets in DI water. The pH was monitored using a Denver Instrument UltraBasic Benchtop pH meter (Bohemia, NY), and solvents were filtered using 0.2 μm filters (Whatman, GE Healthcare or Durapore, Merck Milliproe) prior to use.^68^Ga was eluted from a 50 mCi ^68^Ge/^68^Ga generator (iThemba LABS, South Africa) and purified according to reported methods (Lin et al. 2015). ^67^Ga-citrate was purchased from Isologic (Burlington, Canada) and also purified following the same procedures. The activity was measured using a Capintec (Ramsey, NJ) CRC^®^-25R/W dose calibrator.

### Binding affinity

The binding affinity of ^nat^Ga-labeled compounds, ^nat^Ga-DOTATOC, ^nat^Ga-DOTATATE and ^nat^Ga-NODAGA-JR11 to sstr2 was determined using a membrane-based competition binding assay. Somatostatin-28 (SRIF-28) purchased from Bachem (Torrance, CA) was used as a known positive control for affinity determination. Purified Chinese hamster ovary-K1 (CHO-K1) membranes overexpressing human sstr2 (Perkin Elmer, Waltham, MA) were incubated with ^125^I-[Tyr^11^]-somatostatin-14 (^125^I-[Tyr^11^]-SRIF14, Perkin Elmer) and competing non-radioactive ligand in a 96-well, 1.2 μm glass fibre filter plate (EMD Millipore, Darmstadt, Germany). Prior to assay, the plate filters were pre-soaked in 0.1% polythylenimine (Sigma, St. Louis, MO) for 1 h at ambient temperature. Following pre-incubation, membranes (25 μg/well), ^125^I-[Tyr^11^]-SRIF14 (0.05 nM) and various concentrations of competing peptides (10 μM to 1 pM) were diluted in assay buffer (25 mM HEPES, pH 7.4, 10 mM MgCl_2_, 1 mM CaCl_2_, 0.5% BSA) and incubated for 1 h at 27 °C with moderate shaking. Once complete, the incubation mixture was aspirated through the filters, followed by 6 washes with 200 μL ice-cold wash buffer (50 mM Tris–HCl pH 7.4, 0.2% BSA). Each filter was removed and counted on a PerkinElmer WIZARD 2480 gamma counter. The inhibition constant (K_i_) was calculated by fitting the data to a one-site Fit-Ki curve in GraphPad Prism v7.02. To ensure that the concentration of our peptides, and hence our determination of K_i_, was accurate, the peptide concentration was determined by amino acid analysis at the SPARC BioCentre (Toronto Hospital for Sick Children, Toronto, Canada), where peptides were hydrolyzed and comprising amino acids were separated on HPLC. The peptide content was calculated by comparing the concentration of selected amino acids to known standards.

### Radiolabeling

^68^GaCl_3_ in 0.5 mL DI water was added into an 8 mL glass vial preloaded with 25 μg (30 μg for NODAGA-JR11) peptide precursor and HEPES buffer solution (0.7 mL, pH 5). The vial was sealed with a screw cap and heated in a microwave oven (catalog number: DMW7700WDB, Danby Appliance, Findlay, Ohio) as described previously (Lin et al. 2015). Heating time was 60 s, and the microwave power level was set to “2.” Reaction temperature was not determined. The reaction mixture was cooled, and directly injected into the HPLC semi-preparative column (4.5 mL/min) for purification. HPLC conditions, buffers and retention times are described in Table 1. The ^68^Ga-labeled peptide was collected and diluted with 50 mL 0.05 M ammonium formate solution and passed through a C18 light Sep-Pak cartridge. The product was eluted with 90% ethanol in saline and formulated in saline for animal studies. The Sep-Pak purification was performed to remove HPLC solvents (especially MeCN), concentrate the product, and formulate the tracer in solution suitable for injection into mice. Quality control was done using the analytical column (2 mL/min) and the same conditions described in Table 1. Specific activity was calculated via dividing the radioactivity injected into HPLC (analytical column) by the mass of the tracer. The mass of the radiotracer was calculated based on UV absorbance from a standard curve, constructed using serial dilutions of corresponding ^nat^Ga cold-standard. Radiolabeling with ^67^Ga for saturation binding assays was performed according to the same procedures for the preparation of their ^68^Ga analogs.

**Table 1:** HPLC conditions and retention times (*t*_R_)

Tracer	HPLC conditions	*t*_R_ on semi-preparative column	*t*_R_ on analytical column
^68^Ga-NODAGA-JR11	77% / 23% PBS/ MeCN	14.3 min	5.3 min
^68^Ga-DOTATOC	79% / 21% PBS / MeCN	19.2 min	6.8 min
^68^Ga-DOTATATE	81% / 19% TEAP / MeCN	20.4 min	7.4 min

### Cell culture

All imaging and biodistribution studies were performed using the human breast carcinoma, ER-positive cell model ZR-75-1 purchased from ATCC (Manassas, VA). In addition, HeLa cells, Jurkat cells (ATCC) and sstr5-transfected HEK-293 cells (HEK-sstr5, gifted from Dr. Stefan Schultz, Universitaetsklinikum, Jena, Germany) were used for quantitative-PCR (qPCR) standard curve construction. ZR-75-1 cells were cultured in RPMI 1640 + GlutaMAX^TM^ media purchased from Life Technologies (Carlsbad, CA) and supplemented with 10% FBS from VWR Life Science Seradigm (Radnor, PA). Jurkat cells were grown in the same base media, and contained 10% heat-inactivated FBS and 1 mM sodium pyruvate. HeLa cells were grown in DMEM + GlutaMAX^TM^ (Life Technologies) with 10% FBS. HEK-sstr5 cells were grown in DMEM + GlutaMAX^TM^ with 10% FBS, and contained 0.5 mg/mL G418 to maintain sstr5 expression. All cell cultures were exposed to 100 I.U./mL penicillin/streptomycin (Life Technologies) and grown in a humidified atmosphere at 37 °C with 5% CO_2_.

### Quantitative-PCR

The transcriptional expression of sstr1, sstr2, sstr3, sstr4 and sstr5 in ZR-75-1 cells was determined relative to reference gene hypoxanthine phosphoribosyltransferase 1 (HPRT1) using qPCR. Total RNA from ZR-75-1 cells was purified using the GenElute^TM^ Mammalian total RNA miniprep kit (Sigma), treated with amplification grade DNase I (Sigma), and measured using a NanoDrop^TM^ spectrophotometer. 2.0 μg of total ZR-75-1 RNA was reverse transcribed in a 20 μL reaction using SuperScript® VILO^TM^ cDNA synthesis kit from Invitrogen (Carlsbad, CA). qPCR was set up in 384-well plates, in a total volume of 10 μL; each reaction containing 1 μL template cDNA, 500 μM forward and reverse primers, 250 μM probe, and 1X SsoAdvanced^TM^ universal probes supermix from Bio Rad (Hercules, CA). Each reaction was performed in triplicates and repeated 3 times. Predefined primers (forward and reverse) and probes for all six genes were purchased from Integrated DNA Technologies (Coralville, Iowa); see Additional file 1 for assay names. The Quantstudio^TM^ 6 K Flex Real-Time PCR system from Thermo Fisher (Carlsbad, CA) was used for amplification and detection. The concentration of each target was determined by interpolating the C_t_ value from respective standard curves of known concentrations. To construct the standard curves, RNA from cell lines with known expression of sstr subtypes was purified and reverse-transcribed as described above. Target sstr genes were PCR amplified using Q5® high-fidelity DNA polymerase (New England BioLabs, Ipswich, MA) as per the manufacturer’s instructions, using the same primers as the qPCR reactions (without the fluorogenic probe). PCR products were separated on a 2% agarose gel, and target bands were extracted and purified using the Monarch® DNA gel extraction kit (New England BioLabs). The amount of DNA was quantified using Qubit® dsDNA HS assay kit (Thermo Fisher) and the number of copies/μL was calculated using the following formula:

$$ \mathrm{copies}/\upmu \mathrm{L}=\frac{\mathrm{DNA}\ \mathrm{Concentration}\ \left(\mathrm{g}/\upmu \mathrm{L}\right)}{\mathrm{amplicon}\ \mathrm{length}\ \left(\mathrm{bp}\right) \times 650\ \mathrm{g}/\mathrm{mol}} \times 6.022\ \mathrm{n}1{0}^{23}\mathrm{copies}/\mathrm{mol} $$

Standard curves were constructed from 10-fold serial dilutions (10^5^ copies/μL to 1 copy/μL) and assayed by qPCR in triplicates. All standard curves were repeated 2–3 times. Sstr1 transcripts were amplified from HeLa cells, sstr2 from ZR-75-1 cells, sstr3 from Jurkat cells, sstr4 from the Chantest^TM^ human sstr4 receptor cell line (irradiated cells) from Charles River Laboratories (Wilmington, MA), and both sstr5 and HPRT1 from HEK-sstr5 cells. Representative standard curves, primer and probe information, and cycling conditions for both PCR and qPCR can be found in the Additional file 1.

### Saturation binding assays

Saturation binding assays were performed in vitro on ZR-75-1 cells using variable concentrations (0.1 – 100 nM) of ^67/nat^Ga-labeled tracers. Cells were grown to near-confluence in 12-well plates, and growth media was replaced with reaction media (RPMI, 1% BSA, 100 I.U./mL penicillin/streptomycin) 1 h before the assay. Cells were treated in duplicates with ^67/nat^Ga-DOTATOC, ^67/nat^Ga-DOTATATE or ^67/nat^Ga-NODAGA-JR11 in 500 μL reaction media and incubated for 1 h at 25 °C. Excess cold-standard (1.2 μM) was used to block receptors and determine non-specific binding. After the incubation period, the reaction media was aspirated, and the cells were washed 3 times with cold PBS. Cells were lysed and collected with 1 M NaOH and counted in a PerkinElmer WIZARD 2480 gamma counter. The number of binding sites per cell was calculated and fitted to a one-site binding model in GraphPad Prism 7.02 to determine the dissociation constant (K_d_) and number of binding sites (B_max_).

### Estrogen pellet implant and tumor inoculation

All animal studies were done in compliance with the Canadian Council on Animal Care guidelines and were approved by the Animal Care Committee of University of British Columbia (Vancouver, Canada). Immunodeficient female NOD.Cg-*Prkdc* ^*sci*^ *Il2rg* ^*tm1Wjl*^/SzJ mice (NOD *scid* gamma) were obtained from an in-house breeding colony at the Animal Resource Centre of the BC Cancer Agency Research Centre and also from Jackson Laboratory. To sustain the growth of the ER-positive ZR-75-1 cell model, animals were administered a 1.7 mg, 60-day slow-release estrogen pellet from Innovative Research of America (Sarasota, FL) subcutaneously in the dorsal space of the neck. 3–5 days post pellet-implant, 10 million ZR-75-1 cells were re-suspended in a mixture of 1:1 PBS and Matrigel (Corning Inc., Corning, NY) and inoculated subcutaneously on the right shoulder. Tumors were grown for 5–6 weeks, until they reached a size of 7–11 mm in diameter.

### Biodistribution studies

Mice were sedated using 2 mL/min of O_2_ with 2% isoflurane and injected intravenously (i.v.) with 1–2 MBq of ^68^Ga-labeled peptide. Mice were allowed to roam freely for 60 min prior to euthanasia by 2 mL/min of O_2_ with 4% isoflurane followed by CO_2_ asphyxiation. Blood was promptly collected by cardiac puncture and weighted. Internal organs were harvested, rinsed in PBS, patted dry and weighed. Organ uptake was measured either in a WIZARD 2480 gamma counter (Perkin Elmer) or Cobra II auto-gamma counter (Packard), both calibrated with standards of known ^68^Ga activity. Uptake was normalized to the injected dose and to the respective weight of the organ, and expressed as percent injected dose per gram of tissue (%ID/g).

### PET/CT imaging

Tumor-bearing mice were injected i.v. with 8–9 MBq of ^68^Ga-labeled peptide. Static PET images were acquired 55 min post-injection for 10 min using an Inveon microPET/CT scanner (Siemens, Erlangen, Germany) as described previously (Lin et al. 2015). A baseline CT scan was used for localization and attenuation correction. Mice were promptly euthanized after imaging, and biodistribution studies were undertaken as described above.

### Statistics

Statistical analysis was performed using GraphPad Prism v7.02 software. Transcriptional sstr expression results were tested using a one-way ANOVA. Binding affinity, B_max_ values, K_d_ values and in vivo organ uptakes were compared between the three groups using a one-way ANOVA. The difference was considered statistically significant if the *p* value was < 0.05. Non-statistically significant findings were indicated as “ns.”

## Results

### Binding affinity and radiolabeling

**Fig. 1:**
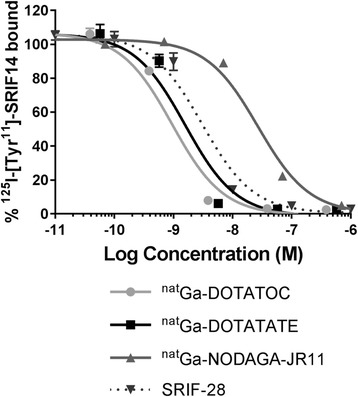
Representative inhibition curves for ^nat^Ga-DOTATOC, ^nat^Ga-DOTATATE, ^nat^Ga-NODAGA-JR11 and SRIF-28 against the binding of ^125^I-[Tyr^11^]-SRIF14 to sstr2-overexpressing CHO-K1 membranes

^nat^Ga-DOTATOC and ^nat^Ga-DOTATATE had an inhibition constant (K_i_) in the low nanomolar range (0.9 ± 0.1 nM, *n* = 4 and 1.4 ± 0.3 nM, *n* = 3 respectively), while the K_i_ of ^nat^Ga-NODAGA-JR11 was higher (25.9 ± 0.2 nM, *n* =3, *p* < 0.001). The SRIF-28 control had a Ki of 3.7 ± 1.7 nM (*n* = 5) in our assays. Representative inhibition curves are shown in Fig. 1. Multiple batches of ^68^Ga-DOTATOC, ^68^Ga-DOTATATE and ^68^Ga-NODAGA-JR11 were prepared in good radiochemical yield (61 ± 5, 62 ± 8 and 68 ± 13% respectively, *n* = 3), purity (>98%) and high specific activity (251.6 ± 33.9, 197.3 ± 85.2 and 138.8 ± 2.6 MBq/nmol respectively, *n* = 3). The particular tracer preparations used for animal studies corresponded to radiochemical yields of 62, 66 and 58% and specific activities of 281.2, 218.3 and 136.9 MBq/nmol for ^68^Ga-DOTATOC, ^68^Ga-DOTATATE and ^68^Ga-NODAGA-JR11 respectively.

### Saturation binding assays

**Fig. 2:**
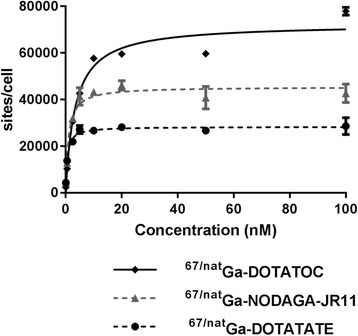
Representative saturation binding curves for ^67/nat^Ga-NODAGA-JR11, ^67/nat^Ga-DOTATOC and ^67/nat^Ga-DOTATATE to ZR-75-1 cells in vitro. The non-specific binding, determined by blocking with excess cold-standard, was subtracted and only specific binding is shown

^67^Ga-labeled peptides used for saturation binding assays were prepared using the same procedures as the ^68^Ga-analogs. ^67^Ga-NODAGA-JR11, ^67^Ga-DOTATOC and ^67^Ga-DOTATATE were labeled in 4, 57, 25% decay-corrected radiochemical yield, > 99% radiochemical purity and 11.5, 392, 444 MBq/nmol specific activity respectively. In vitro saturation binding assays revealed that agonist ^67/nat^Ga-DOTATOC bound to more sites on ZR-75-1 cells (6.64 ± 0.39 × 10^4^ sites/cell) compared to both ^67/nat^Ga-DOTATATE (2.85 ± 0.02 × 10^4^ sites/cell, *p* < 0.001) and ^67/nat^Ga-NODAGA-JR11 (4.39 ± 0.32 × 10^4^ sites/cell, *p* < 0.001). The dissociation constant (K_d_) in these assays was lowest for ^67/nat^Ga-DOTATATE (0.55 ± 0.015 nM), followed by ^67/nat^Ga-NODAGA-JR11 (1.19 ± 0.06 nM, *p* < 0.001) and finally ^67/nat^Ga-DOTATOC (2.70 ± 0.13 nM, *p* < 0.001). See Table 2 for B_max_ and K_d_ values, and Fig. 2 for representative saturation binding curves.

**Table 2:** Saturation binding results for respective radiotracers with ZR-75-1 cells

	^67/nat^Ga-NODAGA-JR11 (*n* = 3)	^67/nat^Ga-DOTATOC (*n* = 3)	^67/nat^Ga-DOTATATE (*n* = 3)
B_max_ (× 10^4^ sites/cell)	4.39 ± 0.32	6.64 ± 0.39	2.85 ± 0.02
K_d_ (nM)	1.19 ± 0.06	2.70 ± 0.13	0.55 ± 0.02

### Transcriptional Sstr expression

**Fig. 3:**
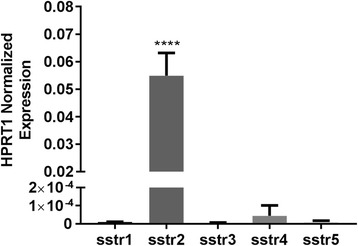
Relative transcriptional expression of sstr subtypes normalized to housekeeping gene HPRT1 (*n* = 3 for each)

We calculated the target gene/HPRT1 copy number ratio for all five somatostatin subtypes and found predominant expression of sstr2. The normalized expression of sstr2 to HPRT1 was 0.055 ± 0.0083 (*n* = 3), and < 0.00005 for all other subtypes (*n* = 3 each, *p* < 0.001) (Fig. 3). For all subtypes except sstr2, we calculated < 10 copies/μL of target transcript in our cDNA preparation. In comparison, we identified 12,915 ± 2218 copies/μL (*n* = 4) HPRT1 transcripts, and 681 ± 148 (*n* = 3) copies/μL sstr2 transcripts.

### Tumor and organ uptake

**Fig. 4:**
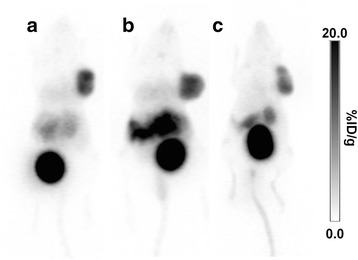
PET maximum intensity projection images of **a**. ^68^Ga-DOTATOC, **b**. ^68^Ga-DOTATATE and **c**. ^68^Ga-NODAGA-JR11 in ZR-75-1 tumor bearing mice

A full overview of tracer biodistribution is presented in Table 3. Among the three tested tracers, we found that the antagonist ^68^Ga-NODAGA-JR11 had the lowest tumor uptake (12.2 ± 0.8%ID/g) compared to agonists ^68^Ga-DOTATOC (18.4 ± 2.9%ID/g, *p* < 0.001) and ^68^Ga-DOTATATE (15.2 ± 2.2%ID/g, ns) (Fig. 4). ^68^Ga-NODAGA-JR11 had tumor-to-blood and tumor-to-muscle ratios of 15.6 ± 2.2 and 45.2 ± 11.6 respectively compared to ^68^Ga-DOTATOC (41.1 ± 5.7, *p* < 0.001 and 171.5 ± 55.3, *p* < 0.01 respectively) and ^68^Ga-DOTATATE (44.7 ± 11.7, *p* < 0.001 and 152.0 ± 60.8, *p* < 0.01 respectively). ^68^Ga-DOTATATE had the highest uptake in non-tumor sstr2-positive organs such as intestines, stomach, pancreas, adrenal glands and lungs, followed by ^68^Ga-DOTATOC and lastly by ^68^Ga-NODAGA-JR11. The excretion profile of all three tracers was predominantly renal, with high uptake in the kidneys and bladder, and low uptake in the liver. The agonist ^68^Ga-DOTATATE had the lowest kidney uptake (8.5 ± 1.7%ID/g), compared to ^68^Ga-DOTATOC (9.3 ± 1.7%ID/g) and ^68^Ga-NODAGA-JR11 (14.1 ± 1.7%ID/g).

**Table 3:** Biodistibution of ^68^Ga-NODAGA-JR11, ^68^Ga-DOTATOC and ^68^Ga-DOTATATE in NOD *scid* gamma ZR-75-1 tumor-bearing mice

	^68^Ga-NODAGA-JR11 *n* = 5		^68^Ga-DOTATOC *n* = 6		^68^Ga-DOTATATE *n* = 6	
	Mean	SD	Mean	SD	Mean	SD
**Tumor**	**12.21**	**0.78**	**18.44**	2.87 ***	**15.22**	**2.20**
Blood	0.80	0.10	0.45	0.09 ***	0.35	0.06 ***
Fat	0.23	0.11	0.18	0.09	0.28	0.15
Ovaries	0.67	0.22	0.51	0.07	0.89	0.35
Uterus	0.86	0.22	0.54	0.11	0.84	0.10
Intestines	0.90	0.20	2.39	0.30 ***	7.35	0.44 ***
Stomach	1.36	0.78	2.75	1.25	8.29	3.06 ***
Pancreas	9.29	2.03	11.01	1.32	51.56	5.47 ***
Spleen	0.39	0.05	0.46	0.11	0.84	0.38
Kidneys	14.12	1.65	9.27	1.73 ***	8.45	1.73 ***
Adrenals	2.00	0.65	9.52	3.78 **	15.20	7.26 **
Liver	0.99	0.12	0.71	0.17	1.95	0.52 ***
Lungs	4.72	0.71	22.93	6.12 ***	28.66	2.94 ***
Heart	0.46	0.06	0.30	0.04 *	0.67	0.13 **
Muscle	0.28	0.07	0.11	0.02 ***	0.11	0.04 ***
Bone	0.26	0.04	0.20	0.03	0.25	0.03
Brain	0.05	0.02	0.03	0.01	0.03	0.01
**Tumor to Background Ratios**						
Blood	15.56	2.20	41.13	5.68 ***	44.65	11.74 ***
Muscle	45.15	11.56	171.51	55.33 **	151.95	60.75 **
Kidneys	0.87	0.12	2.01	0.24 ***	1.88	0.48 ***

Organ uptake is expressed as mean ± standard deviation (SD) in units of percent injected dose per gram of tissue (%ID/g). The tumor uptake is highlighted in bold. Means were statistically compared to the respective organ uptake of ^68^Ga-NODAGA-JR11 and *p* values < 0.05, < 0.01, and < 0.001 were expressed as *, ** and *** respectively

## Discussion

In this study, we used a human breast cancer cell model with endogenous sstr2 expression (ZR-75-1 cells) to compare tumor uptake of the antagonist ^68^Ga-NODAGA-JR11 with two routinely used agonists ^68^Ga-DOTATOC and ^68^Ga-DOTATATE. Most studies evaluating sstr tracers in vivo typically used a rat pancreatic cell model, i.e. AR42J (Froidevaux et al. 2002), or HEK cells transfected with somatostatin receptors (Fani et al. 2012b; Ginj et al. 2006; Fani et al. 2011), which may not adequately represent a breast cancer phenotype. We chose to use the chelator NODAGA instead of DOTA for the antagonist, as ^68^Ga-NODAGA-JR11 showed better binding affinity and higher tumor uptake compared to ^68^Ga-DOTA-JR11 in a preclinical model (Fani et al. 2012b). ^68^Ga-NODAGA-JR11 is a more potent antagonist, and thus a better candidate for comparison with the current gold-standard agonists ^68^Ga-DOTATOC and ^68^Ga-DOTATATE.

We looked at the transcriptional expression of all five sstr subtypes in ZR-75-1 cells in vitro and found predominant expression of sstr2, with negligible expression of the other four subtypes. The HPRT1-normalized expression level was 0.055 ± 0.0083 for sstr2, and < 0.00005 (*p* < 0.001) for sstr1, sstr3, sstr4 and sstr5. Previous studies have reported a strong correlation between sstr mRNA and protein expression, suggesting that transcriptional studies are adequate for profiling this receptor family (Kumar et al. 2005; Wang et al. 2008; Schaer et al. 1997). Breast carcinoma samples typically show varied expression of all five subtypes, often with 2 or 3 subtypes co-expressed on the same sample (Kumar et al. 2005; Schaer et al. 1997). Although sstr1, sstr3 and sstr5 were identified at high levels in a number of cases, sstr2 was the most commonly expressed (Reubi et al. 2001; Kumar et al. 2005; Vikic-Topic et al. 1995; Evans et al. 1997; Schaer et al. 1997).

We tested the binding affinities of the peptides ^nat^Ga-DOTATOC, ^nat^Ga-DOTATATE and ^nat^Ga-NODAGA-JR11 to human sstr2 in a filtration-based, competition binding assay. For the agonists ^nat^Ga-DOTATOC, ^nat^Ga-DOTATATE and SRIF-28, our inhibition constants (K_i_) were 0.9 ± 0.1, 1.4 ± 0.3 and 3.7 ± 1.7 nM respectively. Our K_i_ values are comparable to the IC_50_ values reported by Reubi et al. (K_i_ values were not reported), which were 2.5 ± 0.50, 0.2 ± 0.04 and 2.7 ± 0.30 nM respectively (Reubi et al. 2000). As IC_50_ values are dependent on the concentration of substrates used in a specific assay, they are not reproducible between laboratories, and it is recommended that K_i_ values are calculated. Although ^nat^Ga-DOTATATE was reported to have very high affinity to sstr2 (12 fold higher than ^nat^Ga-DOTATOC) (Reubi et al. 2000), we did not observe this in our experiments. This may explain, in part, why the diagnostic performance of both peptides is similar in clinical studies, with perhaps a slight advantage for ^68^Ga-DOTATOC (Poeppel et al. 2011).

We observed a significantly lower binding affinity for the antagonist ^nat^Ga-NODAGA-JR11 (K_i_ = 25.9 ± 0.2 nM) compared to the two agonists, and also compared to the IC_50_ reported by Fani et al. (IC_50_ = 1.2 ± 0.2 nM) (Fani et al. 2012b). These differences could be partially attributed to differences in methodology and assay conditions. We used a filtration-based binding assay with an SRIF-14 based competing hot ligand (^125^I-[Tyr^11^]-SRIF14), whereas other reports used an autoradiography approach and the SRIF-28 analog: ^125^I-[Leu^8^, DTrp^22^, Tyr^25^]-somatostatin-28 (Fani et al. 2012b). A standardized assay system and a direct comparison between classical protein binding assays and autoradiography methods would be valuable to improve our understanding of the structure-activity relationship of these ligands. When assessing binding affinity based on K_d_ values, we observed a different relationship, more closely resembling that reported in the literature. ^67/nat^Ga-DOTATATE bound sstr2 receptors on ZR-75-1 cells with the lowest K_d_ (0.55 ± 0.02 nM) compared to ^67/nat^Ga-DOTATOC (2.70 ± 0.13 nM) and ^67/nat^Ga-NODAGA-JR11 (1.19 ± 0.06 nM). It appears that ^67/nat^Ga-NODAGA-JR11 binds receptors on ZR-75-1 cells with high affinity, but is not as potent when competing with reference ligand ^125^I-[Tyr^11^]-SRIF14. These findings are interesting and unexpected, and indicate several factors that must be considered when choosing assay conditions to measure affinities.

When tested in vivo, the agonist ^68^Ga-DOTATOC had the highest tumor uptake (18.4 ± 2.9%ID/g) compared to ^68^Ga-DOTATATE (15.2 ± 2.2%ID/g, ns) and ^68^Ga-NODAGA-JR11 (12.2 ± 0.8%ID/g, *p* < 0.001). ^67/nat^Ga-DOTATOC also had the highest B_max_ value in saturation binding assays (6.64 ± 0.39 × 10^4^ sites/cell), even higher than that observed for antagonist ^67/nat^Ga-NODAGA-JR11 (4.39 ± 0.32 × 10^4^ sites/cell, *p* < 0.001). These results contrast recently published reports stating that antagonists can achieve higher tumor uptake by binding more receptor sites (Fani et al. 2012b; Ginj et al. 2006). It is possible that in a cell model where the target G-protein coupled receptor is overexpressed without concomitant overexpression of the associated G-protein, there would be a greater number of receptors in a low-affinity state (unbound by G-protein), and therefore significantly higher B_max_ values observed for antagonists compared to agonists (Kenakin 1997). Overexpression of, not only the receptors, but also the associated G-protein, would be required to have a more representative model. When Ishihara et al. also overexpressed the G-protein in COS cells transfected with secretin receptor, the number of agonists binding sites increased from 1.8% (of the total seen by the antagonist) to 15% (Ishihara et al. 1991). In an endogenous model, such as the one used herein, where most receptors are found in a high affinity state (bound by the G-protein), imaging with antagonists might not be more advantageous. Similar to our studies, Wadas et al. did not observe higher tumor uptake with antagonist ^64^Cu-CB-TE2A-sst2-ANT compared to agonist ^64^Cu-CB-TE2A-Y3-TATE when using the endogenously expressing sstr2 model, AR42J (Wadas et al. 2008). It is also interesting that the agonists ^67/nat^Ga-DOTATOC and ^67/nat^Ga-DOTATATE showed differing number of binding sites (6.64 ± 0.39 and 2.85 ± 0.021× 10^4^ sites/cell respectively) on the ZR-75-1 cells used in this study. This is an unexpected finding, which could explain why both these compounds have comparable tumor uptake clinically, despite differences in binding affinity.

We observed that other sstr2-positive organs such as pancreas, adrenal glands, intestine and stomach had very high uptake with agonist ^68^Ga-DOTATATE compared to the other two radiotracers. This finding can be explained by injected peptide amount, which was lowest for ^68^Ga-DOTATATE (15.6 ± 4.4 pmol/mouse) compared to ^68^Ga-DOTATOC (33.0 ± 33.5 pmol/mouse) and ^68^Ga-NODAGA-JR11 (40.3 ± 21.5 pmol/mouse). A biphasic relationship between uptake in receptor-positive organs and injected peptide mass has been reported, with maximum tumor uptake reached between 10 – 100 pmol/mouse (Bernhardt et al. 2003; de Jong et al. 1999; Notni et al. 2016). In other sstr2-positive organs, maximum uptake is achieved at lower peptide doses, presumably due to lower absolute receptor quantities (more regionally concentrated) and therefore lower saturation limits in these organs (Bernhardt et al. 2003; de Jong et al. 1999; Notni et al. 2016). In our studies, low capacity organs (i.e. pancreas, adrenals, intestine and stomach), but not high capacity organs (i.e. tumor), showed elevated uptake with ^68^Ga-DOTATATE, indicating this to be a peptide mass effect.

Although tumor uptake is also influenced by peptide dose, we believe the mass differences in our studies were not significant enough to cause major differences between the three groups. De Jong et al. showed optimal CA20948 uptake when ^111^In-DOTATOC was injected in 0.5 μg/rat (~30 pmol/mouse), with uptake > 80% of the maximum in the 0.25 – 1 μg/rat range (~15 – 60 pmol/mouse) (de Jong et al. 1999). Similarly Bernhardt et al. showed > 70% of maximum tumor uptake with ^111^In-pentetreotide (GOT1 cell model) when the tracer was injected between 6.4 – 664 pmol/mouse (Bernhardt et al. 2003). Indeed, normalization of peptide content would remove some variability, and enable a more accurate comparison.

Tumor-to-blood and tumor-to-muscle ratios were lower for ^68^Ga-NODAGA-JR11 (15.6 ± 2.2 and 45.2 ± 11.6 respectively) compared to ^68^Ga-DOTATOC (41.1 ± 5.7 and 171.5 ± 55.3 respectively) and ^68^Ga-DOTA-TATE (44.7 ± 11.7 and 152.0 ± 60.8 respectively). ^68^Ga-NODAGA-JR11 had a ~2 fold higher uptake in the blood and muscle compared to the other two agonists, accounting for the lower tumor contrast. These results differ significantly from recently reported clinical data in subjects with neuroendocrine tumors (Nicolas et al. 2016). The improved contrast reported by Nicolas et al. might be caused by a combination of lower specific activity, combined with a lower binding affinity of ^68^Ga-NODAGA-JR11 (^68^Ga-OPS202) to sstr2, which may decrease tracer accumulation in low capacity binding sites (Nicolas et al. 2016). Alternatively, different pharmacokinetic properties between mice and humans may also contribute to these conflicting results.

All three tested peptides had predominant renal clearance. Exogenous estrogen pellets are known to cause hydronephrosis and urine retention (Gakhar et al. 2009; Ingberg et al. 2012), thus we expected higher than normal kidney uptake due to the indirect effects of estrogen.

Beyond diagnosis, sstr2-targeting tracers can also be used therapeutically. Diagnostic somatostatin radiotracers, such as the ones evaluated in this study, can identify breast cancer lesions and monitor response to therapy by PRRT. Treatment of sstr2-positive breast cancer with therapeutic agents such as ^177^Lu-DOTATATE, ^177^Lu-DOTATOC and ^177^Lu-DOTA-JR11 could be particularly valuable to patients that develop resistance to conventional endocrine therapy. The efficacy and safety of ^177^Lu-DOTATATE was demonstrated in an international, multi-centric phase III clinical trial, and showed a potent tumor response and very favourable toxicity in patients with metastatic midgut NETs (NCT01578239) (Strosberg et al. 2016). Similarly, the safety and tolerability of ^177^Lu-DOTA-JR11, also known as ^177^Lu-OPS201, is currently being tested in phase I/II clinical trials for NET patients (NCT02592707).

## Conclusion

We compared the tumor uptake and biodistribution of two well-known agonists and one antagonist in vivo using ZR-75-1 tumors, a human breast cancer xenograft with endogenous sstr2 expression. In this model, the antagonist ^68^Ga-NODAGA-JR11 had the lowest tumor uptake and contrast among the three tracers; a finding that differs significantly from recently published reports. This result may be explained by the use of an endogenously expressing sstr2 cell model, which would have fewer low-affinity binding sites compared to transfected models. More studies are needed to determine if antagonists are better radiotracers for sstr2 breast cancer imaging than agonists, particularly in other breast cancer xenografts, and ultimately in luminal breast cancer patients.

## Additional file

Additional file 1: Supplemental information. **Figure S1.** Representative standard curve for absolute quantification qPCR experiments. **Table S1.** Standard curve parameters. **Table S2.** PCR Cycling conditions. **Table S3.** qPCR Cycling conditions. (DOCX 38 kb)

## Abbreviations

%ID/g: Percent injected dose per gram
^111^In-Pentatrotide: ^111^In-DTPA-D-Phe-octreotide
^125^I-[Tyr^11^]-SRIF14: ^125^I-Tyr^11^-somatostatin 14
ANOVA: Analysis of variance
BSA: Bovine serum albumin
cDNA: Coding DNA
C_t_: Cycle threshold
CT: Computed tomography
DI: Deionized
DMEM: Dulbecco Modified Eagle Medium
DNA: Deoxyribonucleic acid
DOTA: 1,4,7,10-tetraazacyclododecane-1,4,7,10-tetraacetic acid
DOTATATE: DOTA-Tyr^3^-octrotate
DOTATOC: DOTA-Tyr^3^-octrotide
ER: Estrogen receptor
FBS: Fetal bovine serum
HEK: Human embryonic kidney
HEPES: 4-(2-hydroxyethyl)-1-piperazineethanesulfonic acid
HPLC: High performance liquid chromatography
HPRT1: Hypoxanthine phosphoribosyltransferase 1
i.v.: Intravenous
IC_50_: Half maximal inhibitory concentration
K_i_: Inhibition constant
MeCN: Acetonitrile
mRNA: Messenger RNA
^nat^Ga: Natural gallium
NET: Neuroendocrine tumor
NOD scid gamma: NOD.*Cg-Prkdc* ^*scid*^ *Il2rg* ^*tm1Wjl*^/SzJ
NODAGA: 1,4,7-triazacyclononane,1-glutaric acid-4,7-acetic acid
ns: Not significant
PBS: Phosphate buffered saline
PCR: Polymerase chain reaction
PET: Positron emission tomography
PR: Progesterone receptor
PRRT: Peptide receptor radionuclide therapy
qPCR: Quantitative polymerase chain reaction
RNA: Ribonucleic acid
RPMI: Rosewell Park Memorial Institute
SD: Standard deviation
SPECT: Single photon emission computed tomography
SRIF-28: Somatostatin-28
Sstr1-5: Somatostatin receptor subtype 1–5
SUV: Standard uptake value
TEAP: Triethylammonium phosphate
*t*_R_: Retention time
Tris: 2-Amino-2-hydroxymethyl-propane-1,3-diol
