# Supplementary material for: Evaluation of agonist and antagonist radioligands for somatostatin receptor imaging of breast cancer using positron emission tomography
Source: EJNMMI Radiopharm Chem. 2017 Apr 17;2:4. doi: 10.1186/s41181-017-0023-y (PMC5824694; doi:10.1186/s41181-017-0023-y)
Supplement: Supplementary file 1 — Supplemental information. Figure S1. Representative standard curve for absolute quantification qPCR experiments. Table S1. Standard curve parameters. Table S2. PCR Cycling conditions. Table S3. qPCR Cycling conditions. (DOCX 38 kb) [file 41181_2017_23_MOESM1_ESM.docx]

**Evaluation of Agonist and Antagonist Radioligands for Somatostatin Receptor Imaging of Breast Cancer using Positron Emission Tomography**

08

**Fall**

Iulia Dude^1^, Zhengxing Zhang^1^, Julie Rousseau^1^, Navjit Hundal-Jabal^1^, Nadine Colpo^1^, Helen Merkens^1^, Kuo-Shyan Lin^1, 2^, François Bénard*^1, 2^

^1^Department of Molecular Oncology, BC Cancer Agency Research Centre, Vancouver, BC, Canada

^2^Department of Radiology, University of British Columbia, Vancouver, BC, Canada

Corresponding author:

*François Bénard. Address: Department of Molecular Oncology, BC Cancer Agency Research Centre, 675 West 10^th^ Ave, Vancouver, BC V5Z 1L3, Canada. Phone: 604-675-8206. E-mail: fbenard@bccrc.ca.

**SUPPLEMENTAL INFORMATION**

**IDT PrimeTime® qPCR Assays:**

Sstr1: Hs.PT.58.3617180.g

Sstr2: Hs.PT.58.4519773

Sstr3: Hs.PT.58.2857882

Sstr4: Hs.PT.58.25532554.g

Sstr5: Hs.PT.58.25896367.g

HPRT1: Hs.PT.58v.45621572


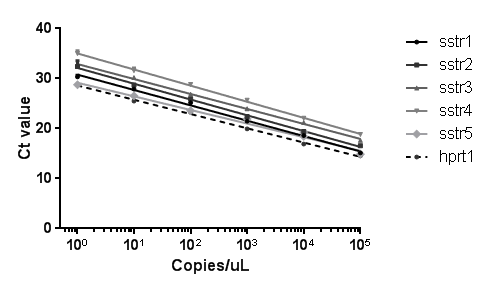


**Supplemental Figure 1**: Representative standard curve for absolute quantification qPCR experiments.

**Supplemental Table 1**: Standard curve parameters

| **Target** | **Efficiency (%)**  **(n=2)** | **Curve Fit (R^2^)**  **(n=2)** |
| --- | --- | --- |
| Sstr1 | 101.6 | 0.9969 |
| Sstr2 | 107.5 | 0.9941 |
| Sstr3 | 112.5 | 0.9937 |
| Sstr4 | 104.7 | 0.9970 |
| Sstr5 | 113.0 | 0.9910 |
| HPRT1 (n=3) | 125.09 | 0.9575 |

**Supplemental Table 2**: PCR Cycling conditions

| **Denaturation** | **98 °C** | **30 sec** |
| --- | --- | --- |
| **40 cycles:** |  |  |
| Denaturation | 98 °C | 10 sec |
| Annealing | sstr1: 60 °C  sstr2: 57 °C  sstr3: 58 °C  sstr4: 57 °C  sstr5: 60 °C  HPRT1: 57 °C | 10 sec |
| Extension | 72 °C | 20 sec |
| **Final Extension** | **72 °C** | **2 min** |
| **Hold** | **4 °C** |  |

**Supplemental Table 3**: qPCR Cycling conditions

| **Hot Start** | **95 °C** | **15 sec** |
| --- | --- | --- |
| **40 cycles:** |  |  |
| Denaturation/  Annealing | sstr1: 60 °C  sstr2: 57 °C  sstr3: 58 °C  sstr4: 58 °C  sstr5: 60 °C  HPRT1: 58 °C | 60 sec |
